# Supplementary material for: Gene expression in soft-shell clam (Mya arenaria) transmissible cancer reveals survival mechanisms during host infection and seawater transfer
Source: bioRxiv. 2024 Sep 19:2024.09.13.612964. Preprint. [Version 1] doi: 10.1101/2024.09.13.612964 (PMC11429866; doi:10.1101/2024.09.13.612964)
Supplement: Supplement 2 [file NIHPP2024.09.13.612964v1-supplement-2.pdf]

bioRxiv preprint doi: https://doi.org/10.1101/2024.09.13.612964; this version posted September 19, 2024. The copyright holder for this preprint (which was not certified by peer review) is the author/funder, who has granted bioRxiv a license to display the preprint in perpetuity. It is made available under aCC-BY 4.0 International license.

SUPPLEMENTAL FIGURES

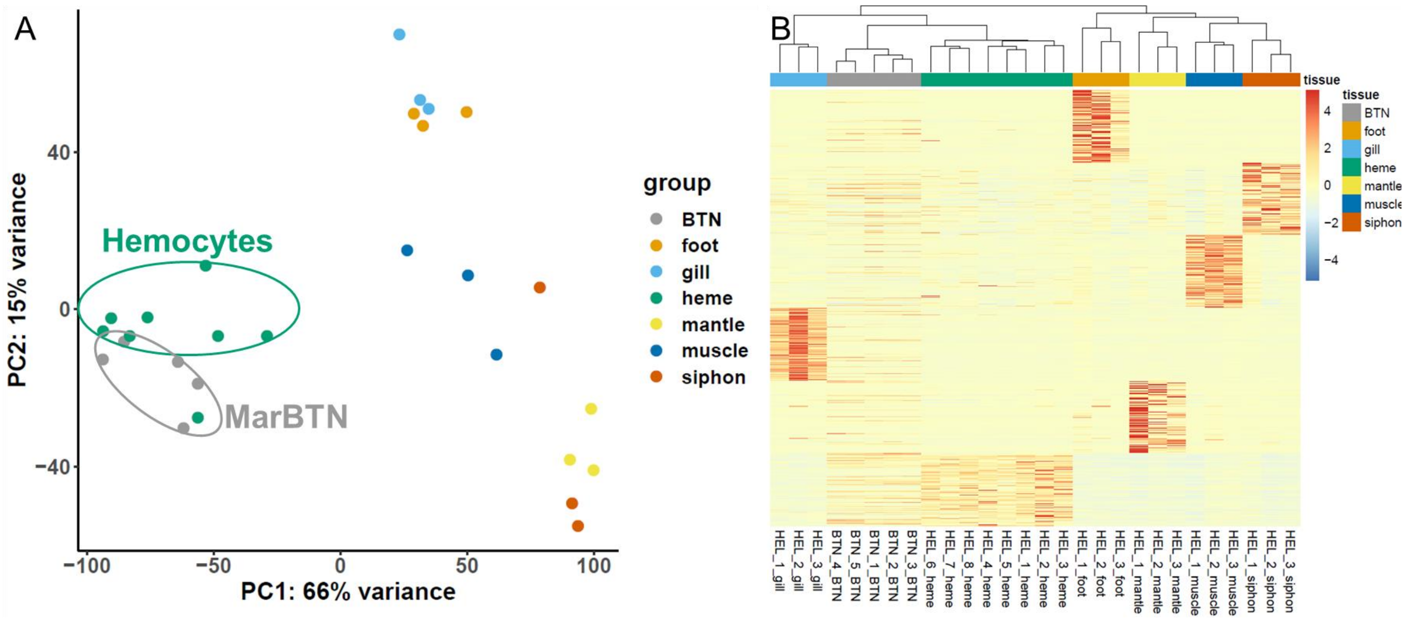

Supplementary Figure 1. Hemocyte origin of MarBTN supported by PCA and clustering with new gene annotations

(A) Principal component analysis of normalized expression across all genes, with PC1 separating MarBTN and hemocytes from all other tissues. **(B)** Hierarchical clustering of all RNA sequenced samples (excluding ASW-treated samples) by the expression of the top 100 most significant genes expressed in each specific healthy tissue relative to all other tissues, with heatmap of normalized relative gene expression for each gene. MarBTN ("BTN") clusters most closely with hemocytes ("heme"), supporting principal component analysis results. Results for both panels closely match similar analyses with previous genome annotation and a smaller sample set [18].

bioRxiv preprint doi: https://doi.org/10.1101/2024.09.13.612964; this version posted September 19, 2024. The copyright holder for this preprint (which was not certified by peer review) is the author/funder, who has granted bioRxiv a license to display the preprint in perpetuity. It is made available under aCC-BY 4.0 International license.

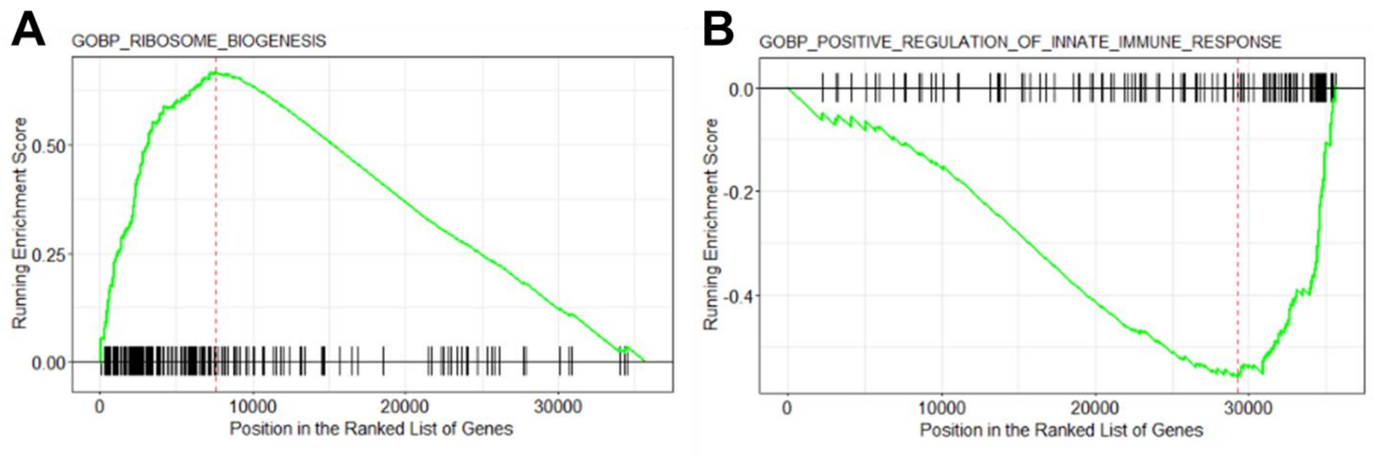

Supplementary Figure 2. Example GSEA results for one each of the top up- and downregulated pathways.

Running enrichment score (green), which increases each time it hits a gene in the gene set (black bars along x-axis) for the ribosome biogenesis biological process (**A**), one of the top upregulated pathways, and positive regulation of innate immune response biological process **(B)**, one of the top downregulated pathways.

bioRxiv preprint doi: https://doi.org/10.1101/2024.09.13.612964; this version posted September 19, 2024. The copyright holder for this preprint (which was not certified by peer review) is the author/funder, who has granted bioRxiv a license to display the preprint in perpetuity. It is made available under aCC-BY 4.0 International license.

1

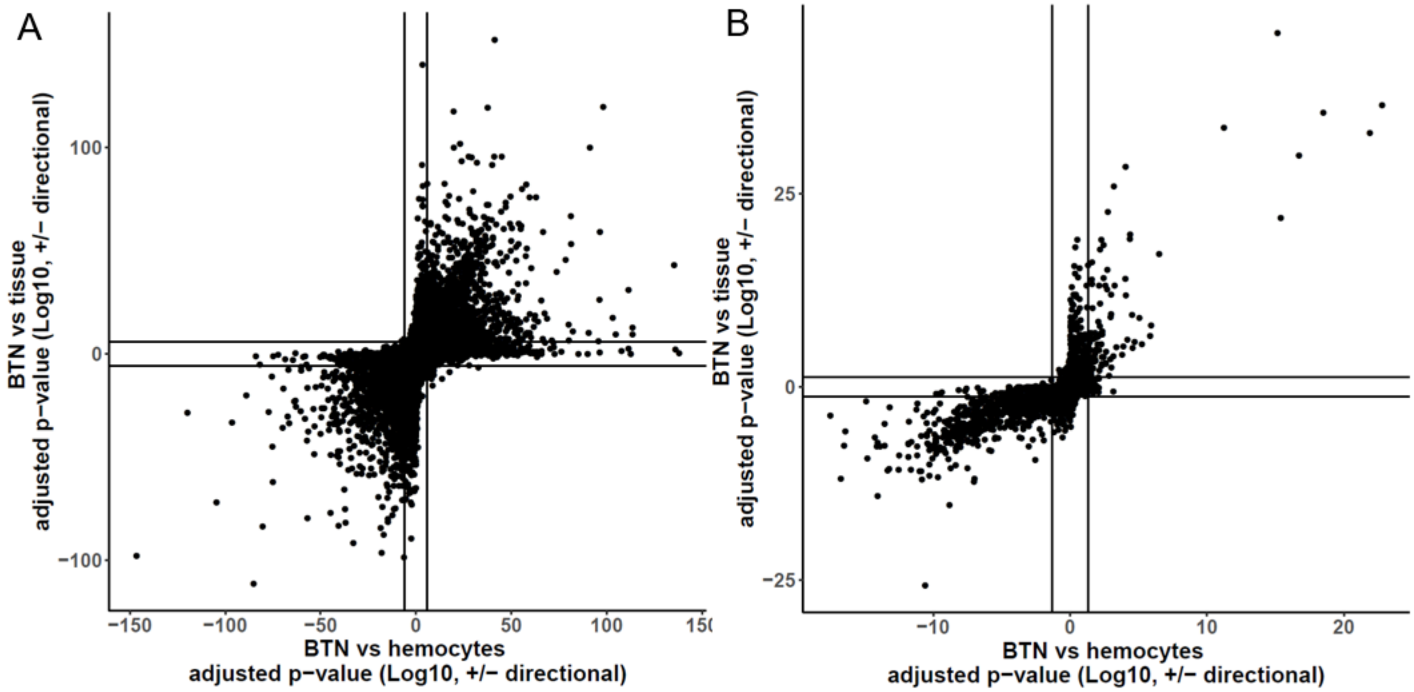

Supplementary Figure 3. Differential expression is similar whether comparing MarBTN to hemocytes or solid tissue.

Adjusted p-values, further adjusted to be positive for upregulation and negative for downregulation, from MarBTN versus healthy hemocytes differential expression results (x-axes) and MarBTN versus solid tissues differential expression results (y-axes) for individual genes **(A)** and gene pathways **(B)**. In general, genes and pathways that are upregulated versus hemocytes are also upregulated versus solid tissues, indicating that major differential expression results and conclusions are not artifacts of the comparison with hemocytes. Lines represent false discovery-corrected $p < 0.05$ significance thresholds.

bioRxiv preprint doi: https://doi.org/10.1101/2024.09.13.612964; this version posted September 19, 2024. The copyright holder for this preprint (which was not certified by peer review) is the author/funder, who has granted bioRxiv a license to display the preprint in perpetuity. It is made available under aCC-BY 4.0 International license.

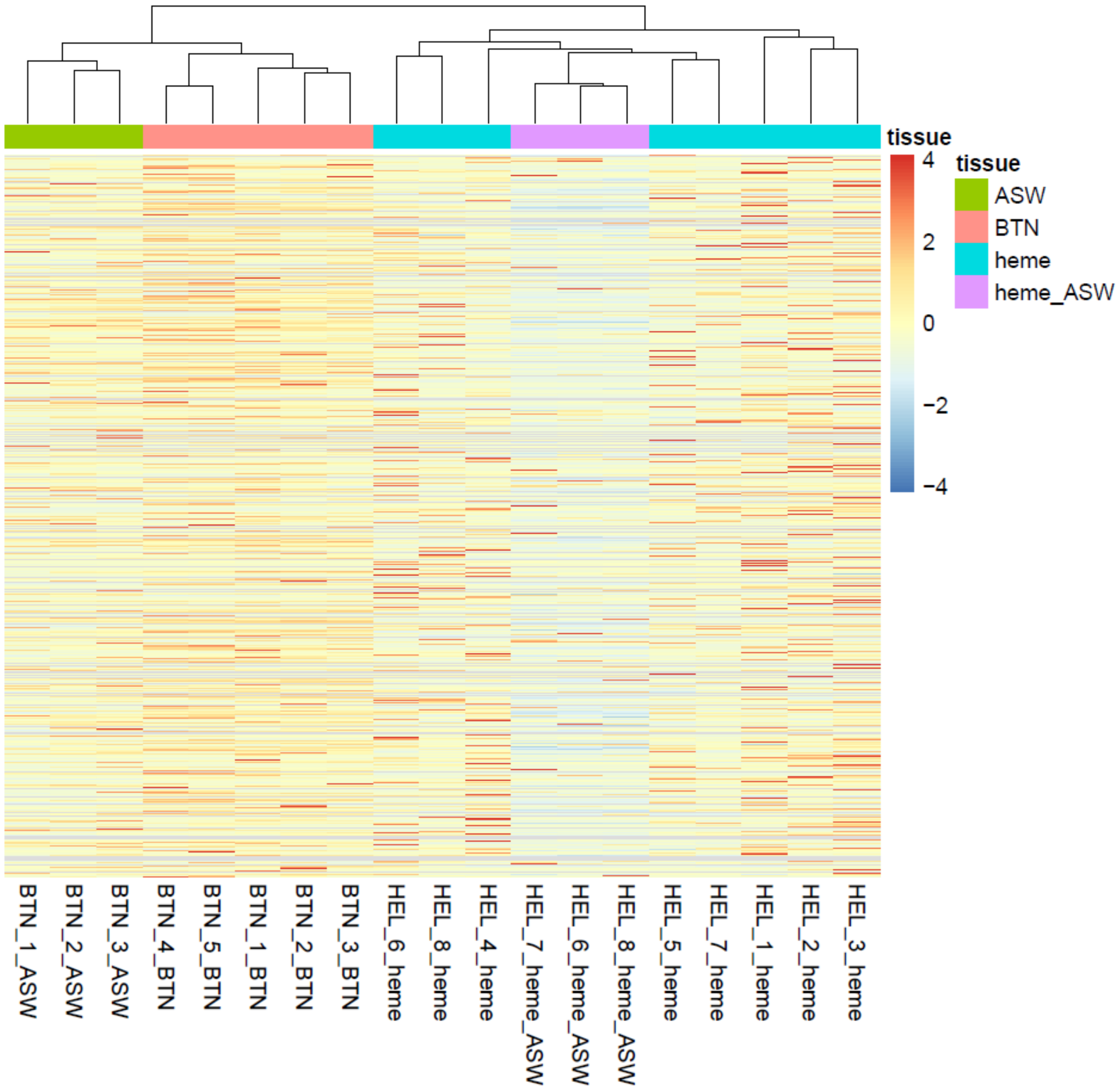

Supplementary Figure 4. Hierarchical clustering separates samples by seawater treatment.

Hierarchical clustering using all genes for untreated healthy hemocytes (“heme” - blue), ASW-treated healthy hemocytes (“heme_ASW” - pink), untreated MarBTN (“BTN” - red) and ASW-treated MarBTN (“ASW” - green). Samples are labeled by their source clam and treatment.

bioRxiv preprint doi: https://doi.org/10.1101/2024.09.13.612964; this version posted September 19, 2024. The copyright holder for this preprint (which was not certified by peer review) is the author/funder, who has granted bioRxiv a license to display the preprint in perpetuity. It is made available under aCC-BY 4.0 International license.

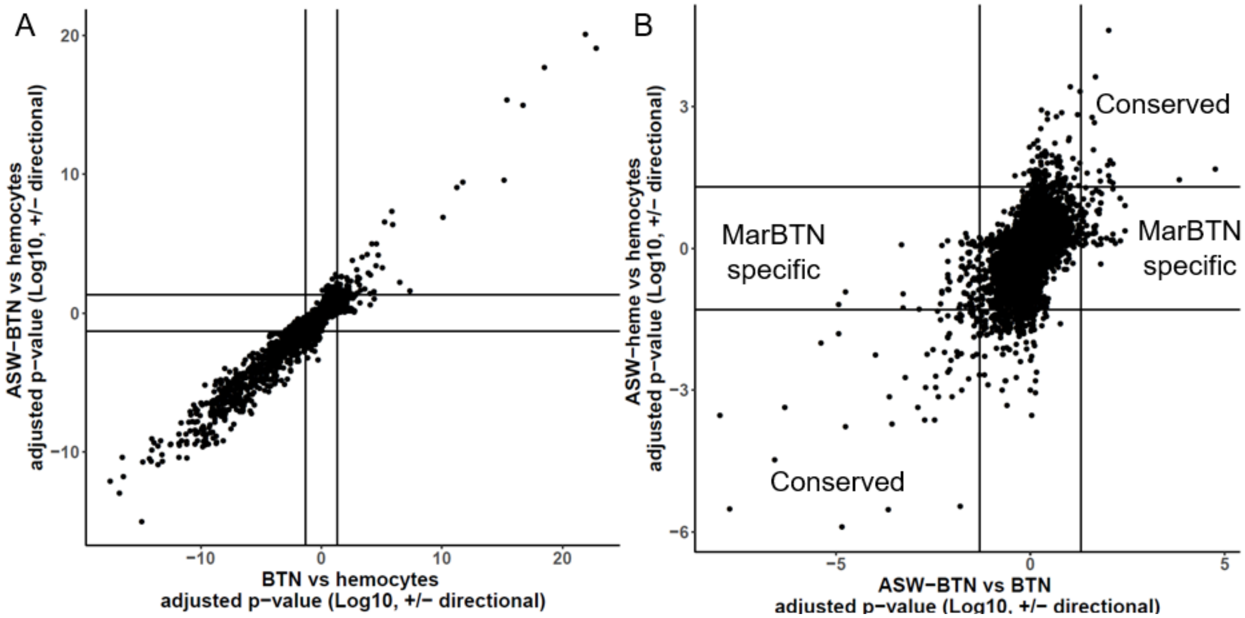

Supplementary Figure 5. Differential expressed pathways show similarities across comparisons.

Adjusted p-values, further adjusted to be positive for upregulation and negative for downregulation, to compare gene set enrichment analysis results from **(A)** ASW-treated MarBTN and untreated MarBTN each versus hemocytes and **(B)** ASW-treated versus untreated for each of MarBTN and hemocytes. In **(A)** results are highly correlated, with the same top up- and downregulated pathways regardless of which treatment is compared to healthy hemocytes. In **(B)**, gene set groupings corresponding to conserved and MarBTN-specific seawater responses from Figure 4 are labeled. Lines represent false discovery-corrected $p < 0.05$ significance thresholds.
